# *Lactobacillus plantarum* Metabolites Elicit Anticancer Effects by Inhibiting Autophagy-Related Responses

**DOI:** 10.3390/molecules28041890

**Published:** 2023-02-16

**Authors:** Sihyun Jeong, Yuju Kim, Soyeong Park, Doyeon Lee, Juho Lee, Shwe Phyu Hlaing, Jin-Wook Yoo, Sang Hoon Rhee, Eunok Im

**Affiliations:** 1 College of Pharmacy, Pusan National University, Busan 46241, Republic of Korea; 2 Research Institute for Drug Development, Pusan National University, Busan 46241, Republic of Korea; 3 Department of Biological Sciences, Oakland University, Rochester, MI 48309, USA

**Keywords:** probiotics, metabolites, *Lactobacillus*, chloroquine, autophagy, tetramethylrhodamine ethyl ester (TMRE)

## Abstract

*Lactobacillus plantarum* (*L. plantarum*) is a probiotic that has emerged as novel therapeutic agents for managing various diseases, such as cancer, atopic dermatitis, inflammatory bowel disease, and infections. In this study, we investigated the potential mechanisms underlying the anticancer effect of the metabolites of *L. plantarum*. We cultured *L. plantarum* cells to obtain their metabolites, created several dilutions, and used these solutions to treat human colonic Caco-2 cells. Our results showed a 10% dilution of *L. plantarum* metabolites decreased cell viability and reduced the expression of autophagy-related proteins. Moreover, we found co-treatment with *L. plantarum* metabolites and chloroquine, a known autophagy inhibitor, had a synergistic effect on cytotoxicity and downregulation of autophagy-related protein expression. In conclusion, we showed the metabolites from the probiotic, *L. plantarum*, work synergistically with chloroquine in killing Caco-2 cells and downregulating the expression of autophagy-related proteins, suggesting the involvement of autophagy, rather than apoptosis, in their cytotoxic effect. Hence, this study provides new insights into new therapeutic methods via inhibiting autophagy.

## 1. Introduction

Colorectal cancer (CRC) is one of the most common types of cancer worldwide in 2020, ranking third in incidence and second in mortality [1,2]. The rising prevalence of CRC is caused by increased meat intake, decreased physical activity, excessive alcohol consumption, and continuous smoking [3,4]. The main causes of death due to CRC are the recurrence and metastasis of cancer cells [5,6,7].

Autophagy is a catabolic recycling process where unnecessary and damaged cellular components are removed, and hence, it is essential for maintaining cellular homeostasis [8,9,10]. Autophagy can be divided into macroautophagy, microautophagy, chaperone-mediated autophagy, and crinophagy [11], with macroautophagy being the most well-characterized. Autophagosomes fuse with lysosomes to form autolysosomes after trapping cellular components for degradation and recycling as substrates in many other places. Therefore, impaired autophagy pathways can affect cellular functions and contribute to the pathogenesis of many diseases [12,13,14].

*Lactobacillus plantarum* (*L. plantarum*), a rod-shaped gram-positive bacterium, is a probiotic that has various beneficial effects on its hosts [15]. In recent decades, with increasing interest in probiotics, *L. plantarum* and its metabolites have emerged as novel therapeutic agents for managing various diseases, such as cancer, atopic dermatitis, inflammatory bowel disease, and infections [16,17,18,19,20,21]. In particular, owing to high mortality rates and globally rising incidences, elucidating the anticancer effects of *L. plantarum* and its metabolites is important for developing novel therapeutics for the treatment of various cancers.

The anticancer effects and its underlying mechanisms of *L. plantarum* and other *Lactobacillus* species have been previously reported [22,23,24,25]. *L. salivarin* REN suppressed the upregulation of COX-2 expression and therefore, decreased the rate of oral carcinogenesis [26,27]. *L. hilgardii* also showed an anticancer effect via inducing apoptosis pathways [22]. Moreover, *Lactobacillus* species are known to regulate autophagy. For instance, *L. casei* ATC 393, *L. rhamnosus* GG, and *L. reuteri* ZJ617 have been reported to regulate autophagy [26]. *L. casei* ATC 393 alleviated dysregulated autophagy induced by H_2_O_2_ via activating the Nrf2-signaling pathway and reducing MMP reduction and intestinal permeability [28]. *L. rhamnosus* GG increased phosphorylation of EGFR and Akt and decreased autophagy activity in *Salmonella Infantis*-infected conditions [29]. *L. rhamnosus* GG also increased T cell maturation and cell proliferation and thus, downregulated the production of IL-2, IL-4, and IL-10 [30]. *L. reuteri* ZJ617 increased the tight junction and decreased MAPK, NFκB, and autophagy-signaling pathways [31].

Many studies have reported *L. plantarum* effectively suppressed the growth of resistant CRC cells [25,32]. Most of the mechanisms that explain the anticancer effect of *L. plantarum* have been with a focus on apoptosis. However, other *Lactobacillus* species have shown to regulate autophagy [22,33,34]. Therefore, we hypothesized the anticancer effect of *L. plantarum* is related to autophagy regulation. The objective of this study is to investigate the autophagy-related anticancer effect of *L. plantarum* metabolites in colorectal adenocarcinoma cells. We aimed to test whether *L. plantarum* metabolites reduce the viability of colon cancer cells and to investigate the inhibitory effects of *L. plantarum* metabolites on the expression of autophagy-related proteins and mitochondrial dysfunction.

## 2. Results

### 2.1. L. plantarum Metabolites Reduced the Cell Viability of Caco-2 Human Colorectal Cancer Cells

**Figure 1:**
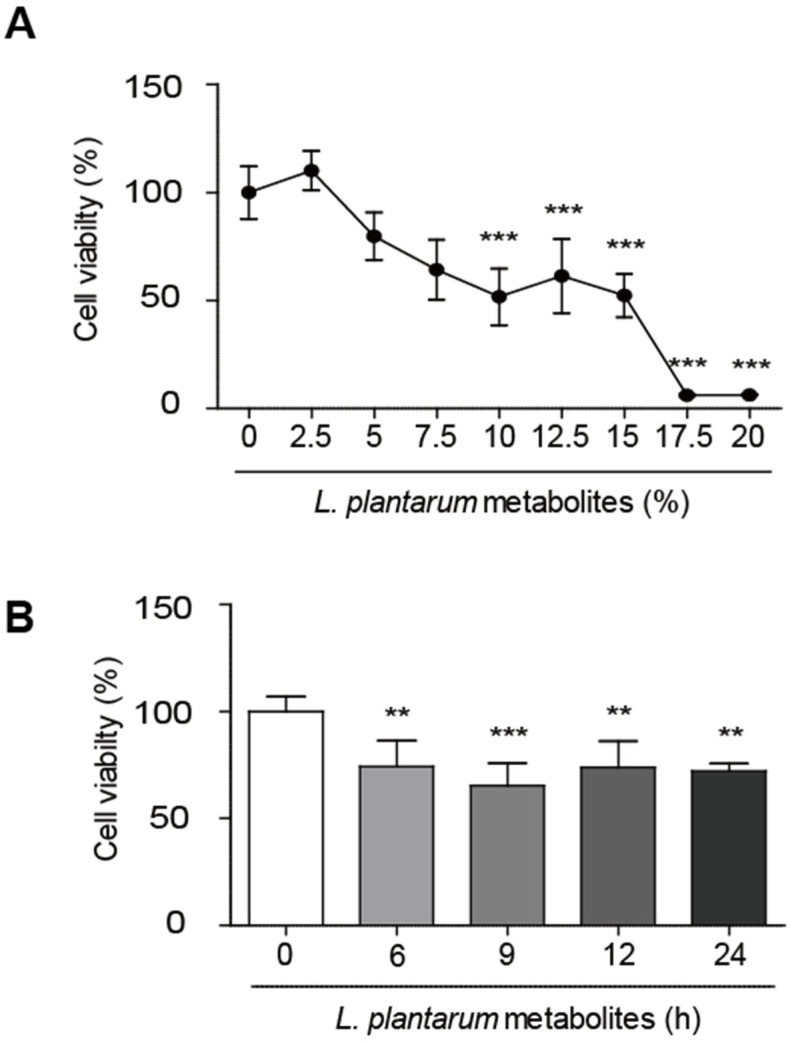
*Lactobacillus plantarum* metabolites decreased the viability of human colon cancer cells. (**A**) Caco-2 cells were treated with *L. plantarum* metabolites at various concentrations from 2.5% to 20% for 24 h. Cell viability was detected using 3-(4,5-dimethylthiazol-2-yl)-2,5-diphenyl tetrazolium bromide (MTT) (0.5 mg/mL) assays. (**B**) Caco-2 cells were treated with a 10% dilution of *L. plantarum* metabolites from 6 h to 24 h. Cell viability was detected using an MTT assay. Data are presented as the mean ± SD. ** *p* < 0.01 and *** *p* < 0.001 compared to untreated control; one-way ANOVA was followed by Tukey’s post hoc test.

An MTT assay was used to investigate the effects of varying concentrations of *L. plantarum* metabolites on the viability of Caco-2 cells. *L. plantarum* metabolites were diluted with Caco-2 culture media without antibiotics at various ratios, and Caco-2 cells were treated with the diluted *L. plantarum* metabolites for 24 h. As shown in Figure 1A, *L. plantarum* metabolites exhibited no cytotoxicity at concentrations up to 5%. Cell viability was reduced significantly from 10% dilution of *L. plantarum* metabolites, and IC50 of *L. plantarum* metabolites was about 10.03%. Caco-2 cells were also treated with *L. plantarum* metabolites from 6 h to 24 h; as seen in Figure 1B; cell viability was significantly decreased in all *L. plantarum* metabolites-treated groups.

**Figure 2:**
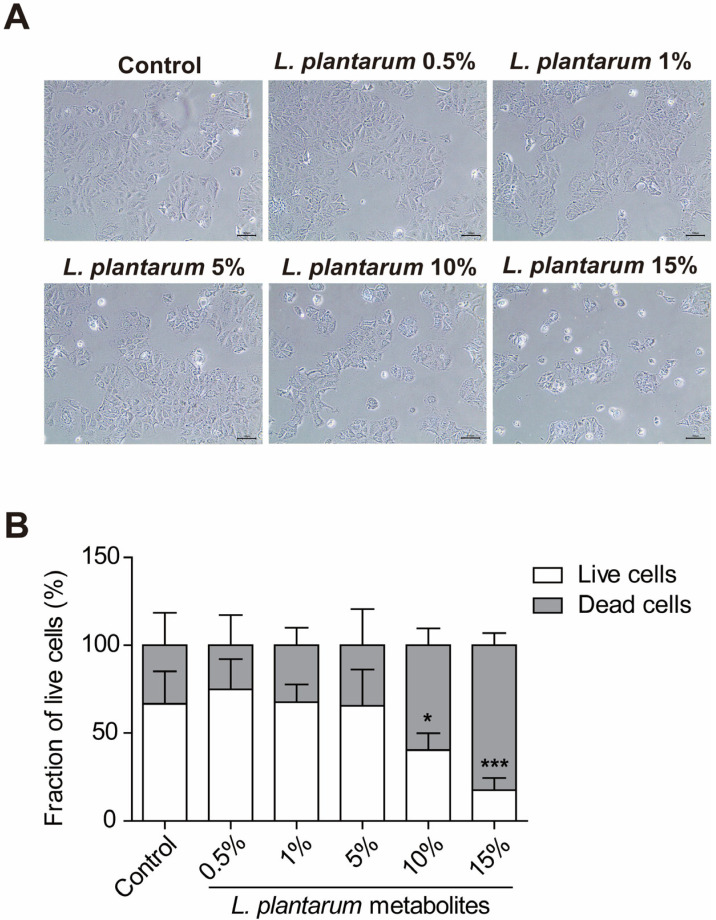
*Lactobacillus plantarum* metabolites decreased the survival of colon cancer cells in a concentration-dependent manner. Caco-2 cells were treated with *L. plantarum* metabolites at concentrations ranging from 0.5% to 15% for 24 h. (**A**) All images were obtained using an MIC-Eyecam-3Mpixel microscope digital camera at 100× magnification. Scale bar, 100 μm. (**B**) The graph indicated the fraction of live cells. Data shown are mean ± SD. * *p* < 0.05, *** *p* < 0.001 compared to live cells with control group with two-way ANOVA.

Cell viability was also analyzed using microscopic imaging, as shown in Figure 2A (100× magnification). Caco-2 cells were treated with various concentrations of *L. plantarum* metabolites for 24 h again. There were no noticeable differences in the 5% *L. plantarum* metabolite-treated group compared to the untreated control group. However, the fraction of live cells decreased in the 10% *L. plantarum* metabolite-treated group significantly, and almost all cells died after treatment with 15% *L. plantarum* metabolites, as seen in Figure 2B. Therefore, we set the *L. plantarum* metabolite concentration to 10% in the succeeding experiments.

### 2.2. L. plantarum Metabolites Downregulated the Expression of Autophagy Markers in Caco-2 Cells

**Figure 3:**
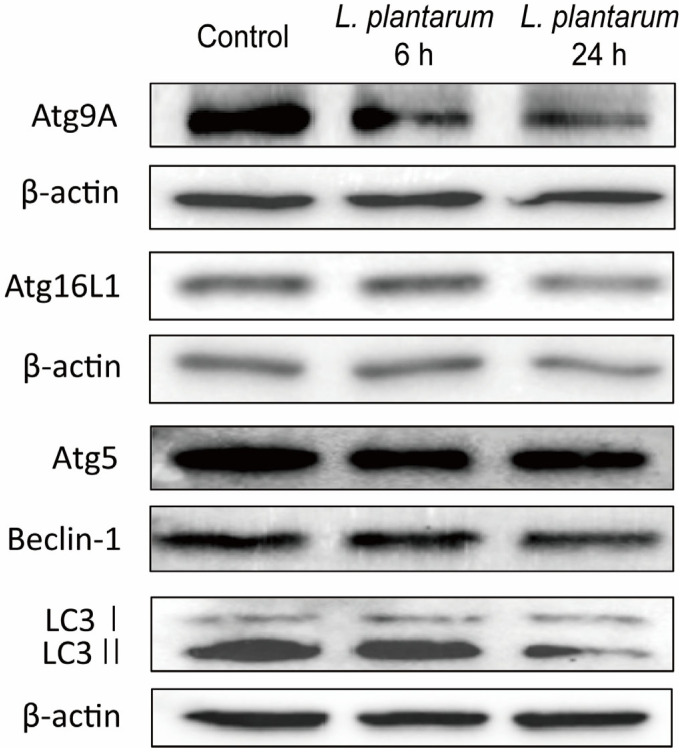
*Lactobacillus plantarum* metabolites suppressed the expression of autophagy-related proteins in colon cancer cells. Caco-2 cells were treated with a 10% dilution of *L. plantarum* metabolites for 6 or 24 h. Representative western blot band images of Atg9A, Atg16L1, Atg5, Beclin-1, LC3 I/II, and β-actin expression in Caco-2 cells are presented. β-actin is used as a loading control.

The expression of autophagy markers, such as Atg9A, Atg16L1, Atg5, Beclin-1, and LC3 I/II, was determined to investigate the effect of *L. plantarum* metabolites on autophagy. Western blot analyses showed the levels of Atg9A, Atg16L1, Atg5, and Beclin-1 decreased following treatment with 10% *L. plantarum* metabolites. The expression of autophagy markers was further reduced in the 24 h-treated group than in the 6 h-treated group. The accumulation of LC3 to LC3 II was decreased in the group treated with *L. plantarum* metabolites for 24 h (Figure 3).

### 2.3. L. plantarum Metabolites Enhanced the Autophagy Inhibition Ability of CQ in Caco-2 Cells

To verify the inhibitory effect of the *L. plantarum* metabolites on autophagy, we used chloroquine (CQ), a well-known autophagy inhibitor, and measured the expression levels of Atg9A, LC3 I/II, Atg5, Atg16L1, and Beclin-1. During autophagy, the autophagosome engulfs cytoplasmic components, and the cytoplasmic form of LC3 I is transformed into LC3 II, which is then recruited to the autophagosome membrane. When the autophagosome fuses with the lysosome and components within the autophagosome are degraded, LC3 II is also degraded [35]. Atg5 is involved in the expansion of the phagophoric membrane in autophagic vesicles and after activation, forms a complex with Atg16L1 to perform its function. Atg5 is also required for the formation of the LC3 I/II complex [36]. Atg9A contributes to membrane growth of the autophagosome [37]. Beclin-1 plays an important role in the regulation of autophagy and apoptosis by interacting with Bcl-2 or PI3K [38].

**Figure 4:**
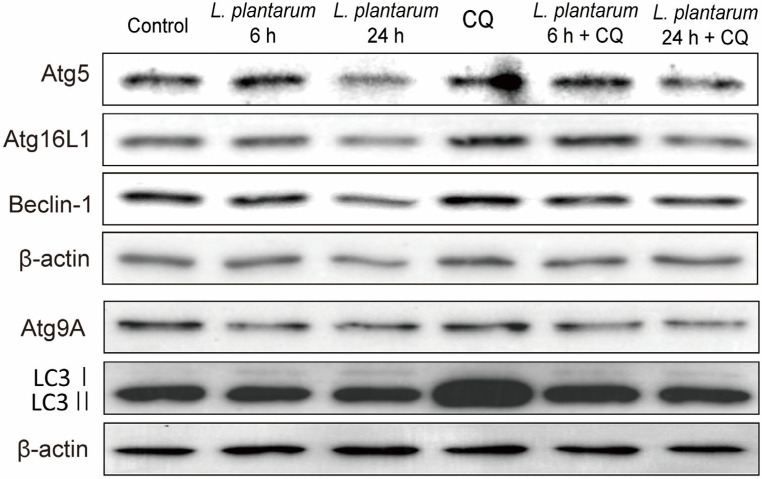
Co-treatment with *Lactobacillus plantarum* metabolites further suppressed the expression of autophagy-related proteins in colon cancer cells than treatment with chloroquine (CQ) alone. Caco-2 cells were pretreated with a 10% dilution of *L. plantarum* metabolites for 6 or 24 h and then treated with CQ (100 μM) for 45 min before harvesting the cell extracts. The protein levels of Atg9A, LC3 I/II, Atg5, Atg16L1, Beclin-1, and β-actin were then observed using western blot analysis. β-actin is used as a loading control.

As before, Caco-2 cells were treated with *L. plantarum* metabolites for 6 or 24 h, and 100 μM CQ was added 45 min before harvesting the cells. CQ is known to block autolysosome formation, leading to an increase in LC3 II and other autophagy marker proteins, while *L. plantarum* metabolites decreased autophagy protein expression (Figure 3 and Figure 4). Co-treatment with *L. plantarum* metabolites and CQ may balance the protein expression levels compared to *L. plantarum* metabolites alone. The protein level of Atg9A decreased in the *L. plantarum* metabolites and CQ co-treatment groups compared to *L. plantarum* metabolites alone. The expression levels of Atg5, Atg16L1, and Beclin-1 slightly decreased in the *L. plantarum* metabolites alone treatment group and the *L. plantarum* metabolites and CQ co-treatment groups. CQ-mediated LC3 II accumulation decreased in the *L. plantarum* metabolites and CQ co-treatment groups (Figure 4).

### 2.4. Co-Treatment with L. plantarum Metabolites and CQ Decreased Caco-2 Cell Viability More than CQ Treatment Alone

**Figure 5:**
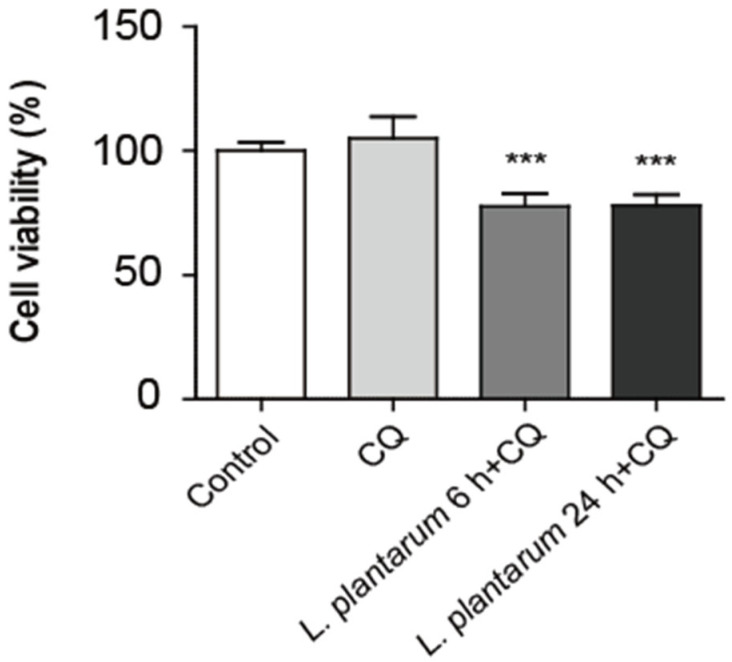
Co-treatment with *Lactobacillus plantarum* metabolites and CQ further decreased the viability of colon cancer cells than treatment with CQ alone. Caco-2 cells were treated with a 10% dilution of *L. plantarum* metabolites for 6 or 24 h and then treated with CQ (100 μM) for 45 min before harvesting the cells. Cell viability was detected using an MTT assay. Data are presented as the mean ± SD. *** *p* < 0.001; one-way ANOVA was followed by Tukey’s post hoc test.

The MTT assay was used to determine the difference between the *L. plantarum* metabolites treatment time and the effects of CQ treatment on the viability of Caco-2 cells. Here, 10% *L. plantarum* metabolites and 100 μM CQ were used to treat Caco-2 cells for 45 min. There was no significant difference between the control and CQ treatment-only groups. Cell viability was decreased upon co-treatment with *L. plantarum* metabolites and CQ, regardless of treatment time (Figure 5).

### 2.5. L. plantarum Metabolites Induced Mitochondrial Dysfunction in Caco-2 Cells

**Figure 6:**
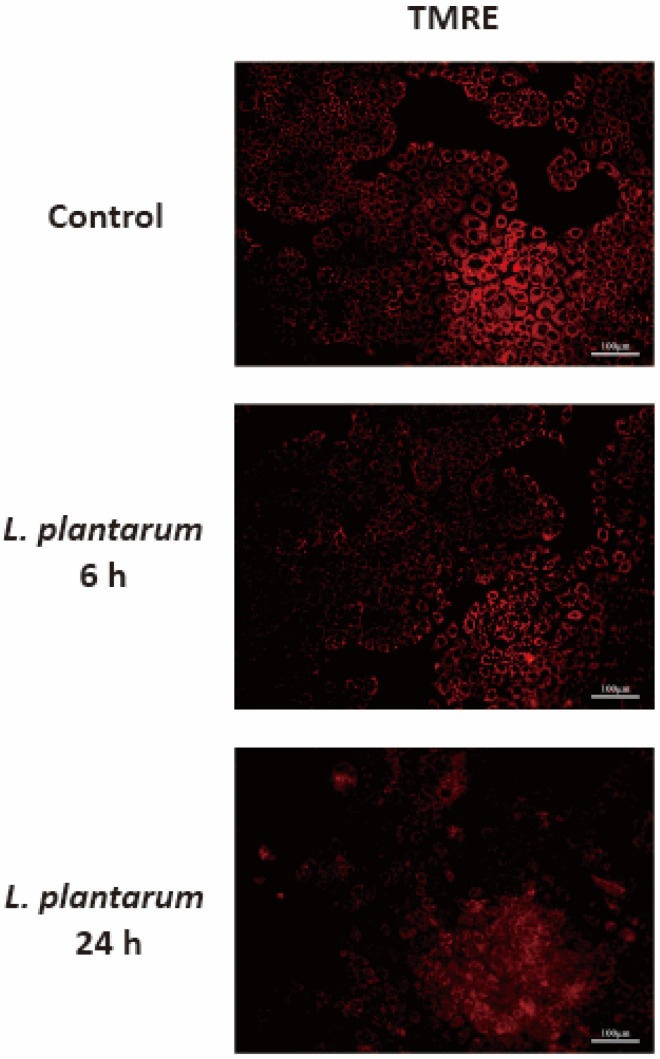
*Lactobacillus plantarum* metabolites induced mitochondrial dysfunction in colon cancer cells. Caco-2 cells were treated with *L. plantarum* metabolites for 6 or 24 h and then stained with tetramethylrhodamine ethyl ester (TMRE, 0.05 μM) for 20 min. The presented images were obtained using a fluorescence microscopy with the same conditions and settings. Scale bar, 100 μm.

Impaired autophagic activity is related to mitochondrial dysfunction. tetramethylrhodamine ethyl ester (TMRE) staining was performed to investigate whether autophagy inhibition induced by *L. plantarum* metabolites was associated with mitochondrial dysfunction. The mitochondrial membrane potential of Caco-2 cells was determined using a fluorescence microscopy. The intensity of TMRE was reduced in cells treated with *L. plantarum* metabolites for 6 h and further reduced in the group treated for 24 h. This implies the mitochondrial membranes of Caco-2 cells were depolarized by the *L. plantarum* metabolites (Figure 6).

## 3. Discussion

Metabolic shift is a major hallmark of cancer [39]. Cancer cells require large amounts of ATP and change their metabolism from oxidative phosphorylation to aerobic glycolysis [40,41]. Metabolic reprogramming in cancer cells is characterized by increased glucose uptake and lactate production, called the “Warburg effect” [42,43]. Reportedly, metabolic reprogramming is also related to chemoresistance, with some reports showing that targeting altered metabolism is effective in overcoming chemoresistance [44,45,46,47]. The chemotherapeutic agent 5-fluorouracil (5-FU) is the most widely used first-line treatment for CRC and is used as a monotherapy or in combination with other anticancer drugs, such as oxaliplatin or irinotecan [48,49]. It has been shown 5-FU is an effective CRC treatment; however, it can induce drug resistance as a side effect [50]. CRC cells that are 5-FU-resistant show cancer stem-cell-like properties and can induce early tumor recurrence or metastasis [51,52]. Therefore, novel therapeutic strategies are needed to overcome chemoresistance and increase the efficacy of anticancer drugs [53,54,55].

The relieving effect of *L. plantarum* on intestinal inflammation has been reported in other studies [56,57]. The gene expression and production of anti-inflammatory factors (IL-10, TGF-β1, and TGF-β2) increased in Caco-2 cells and a DSS-induced mouse model [58,59]. *L. plantarum* reduced the gene expression and production of pro-inflammatory cytokines (IL-1β, IL-6, TNF-α, MPO, and IFN-γ) and suppressed inflammatory responses to normalize the immune barrier [60]. Additionally, in vivo, *L. plantarum* administration reduced the incidence of DSS-induced colitis. In clinical, *L. plantarum* uptake maintained the integrity and permeability of intestinal epithelial cells and increased mucin secretion from goblet cells [61]. Therefore, *L. plantarum* reduced chronic mucosal inflammation and ulcerative colitis symptoms. Chronic intestinal inflammation is important because chronic intestinal inflammation can lead to the development of CRC [62]. Short-chain fatty acids (SCFAs) are also suggested as the main cause for the effect of relieving intestinal inflammation of *L. plantarum*. SCFAs are metabolites of gut microbiota, and *L. plantarum* can also produce SCFAs. SCFAs have immunomodulatory effects and can reduce the expression of pro-inflammatory factors [63]. The reduction of inflammatory responses by SCFAs can effectively contribute to alleviating the symptoms of ulcerative colitis and CRC.

*L. plantarum* and its metabolites have an inhibitory effect on chemoresistant CRC cells, especially 5-FU-resistant CRC cells [32,51,53,54]. *L. plantarum* can exhibit anticancer effects by increasing the cytotoxicity and death of CRC cells or reducing NLRP3 and ERK phosphorylation. *L. plantarum* metabolites can reverse the altered metabolism in cancer, including the reversion of transformed glycolysis to normal glycolysis [6]. Cancer cells require more ATP than normal cells; therefore, their glycolytic rate increases, and they can also undergo aerobic glycolysis [64]. Altered glycolysis increases glucose uptake and lactate production [42] and can promote acidic conditions, one of the hallmarks of cancer as well as chemoresistance [45,65,66,67,68]. However, *L. plantarum* metabolites can reverse altered glycolysis mechanisms, thereby decreasing cancer cell proliferation, increasing apoptosis, and decreasing chemoresistance [53,54]. Thus, *L. plantarum* metabolites can induce therapeutic effects and prevent cancer recurrence.

There have been some reports on the anticancer effects of *L. plantarum* and other *Lactobacillus* strains. For instance, *L. salivarin* REN can decrease the rate of oral carcinogenesis. *L. casei* ATC 393, *L. rhamnosus* GG, and *L. reuteri* ZJ617 have also been reported to show anticancer effects, which are elicited via autophagy-related mechanisms rather than apoptosis [26,28,29,30,31,69]. Many studies reported *L. plantarum* effectively suppresses resistant CRC cells. Most of the mechanisms that explain CRC cell suppression have been described with a focus on apoptosis. Therefore, we conducted experiments focusing on autophagy.

In this study, *L. plantarum* metabolites exhibited cytotoxicity from 10% dilution against colon cancer cell lines, as evidenced by the MTT assay and imaging analysis results. Additionally, a 10% dilution of *L. plantarum* metabolites significantly decreased colon cancer cell viability after treatment from 6 h to 24 h. Treatment with *L. plantarum* metabolites for 6 or 24 h also reduced the expression of autophagy-related proteins, such as Atg9, Atg5, Atg16L1, and Beclin-1. Upon treatment with CQ, a well-known autophagy inhibitor, *L. plantarum* metabolites further reduced expression levels of all four proteins of interest as compared to treatment with CQ alone. Treatment with CQ alone did not decrease the viability of colon cancer cells; however, co-treatment with *L. plantarum* metabolites and CQ significantly decreased cell viability. Using TMRE staining, *L. plantarum* metabolites were shown to depolarize the mitochondrial membranes of colon cancer cells. Further experiments are warranted to investigate the precise role of *L. plantarum* metabolites in each step of the autophagy process and the alteration of autophagy marker expression over time. In addition, it is recommended that *L. plantarum* metabolites are tested and verified in chemoresistant CRC cells.

Whether autophagy promotes or suppresses the growth of cancer cells has not yet been elucidated [68]; however, reports that autophagy inhibition can suppress cancer are highly prevalent [67,70,71,72]. Autophagy can provide the necessary nutrients and energy to cancer cells and increase angiogenesis [73,74]. Cancer cells can easily overcome stress conditions and increase their rate of metastasis and invasion [75,76]. CQ is a well-known autophagy inhibitor, but it is difficult to be taken up by cells in an acidic tumor environment [77]. Therefore, *L. plantarum* is recommended as a novel agent to overcome chemoresistance by inhibiting autophagy. Moreover, *L. plantarum* metabolites can increase the suppressive activity of anticancer agents.

Autophagy is a complex process composed of several sequential steps [78]. Our western blot analysis data suggest *L. plantarum* metabolites and co-treatment with CQ regulate different stages of autophagy. CQ is known to impede the formation of autolysosome, causing an increase in LC3-II and other autophagy marker proteins. Our findings indicate an elevated expression of LC3-II and other autophagy makers after CQ treatment. On the other hand, *L. plantarum* metabolites appear to obstruct phagophore and autophagosome formation, including the steps of vesicle nucleation and vesicle elongation, resulting in decreased expression of autophagy proteins. Co-treatment of *L. plantarum* metabolites and CQ may balance the protein expression levels compared to treatment with *L. plantarum* metabolites alone.

CQ has previously been shown to enhance the sensitivity of the anticancer drug cisplatin in cholangiocarcinoma cells by increasing intracellular, particularly mitochondrial, reactive oxygen species mediated by glucose metabolism and reducing the antioxidant capacity of cells [79]. Therefore, it is possible that CQ may amplify the sensitivity of *L. plantarum* metabolites in cancer cells with high autophagy flow. While probiotics, such as *L. plantarum,* are utilized in anticancer therapies, their modes of action are not yet fully understood. Our study suggests a new mechanism by which *L. plantarum* metabolites suppress autophagy to produce an anticancer effect. In the future, we aim to further investigate the exact role of *L. plantarum* metabolites in each step of the autophagy process and to study the alteration of autophagy marker expression over time.

In conclusion, our results demonstrate *L. plantarum* metabolites are effective agents for inhibiting autophagy in colon cancer cells as they decrease cell viability and downregulate autophagy-related protein expression. When co-treated with CQ, *L. plantarum* metabolites were more effective at downregulating autophagy-related protein expressions than CQ alone. In addition, *L. plantarum* metabolites caused mitochondrial dysfunction by depolarizing the mitochondrial membranes. Thus, *L. plantarum* metabolites have the ability to decrease autophagy activity in colonic cancer cells. These results suggest the autophagy inhibition effect of *L. plantarum* could be useful as a new agent for treating cancer cells.

## 4. Materials and Methods

### 4.1. Preparation of L. plantarum Metabolites

*Lactobacillus plantarum* (KCTC 3108) was purchased from the Korean Collection for Type Cultures (KCTC). To activate *L. plantarum*, the bacterial suspension was spread on BactoTM Lactobacilli MRS Broth (MRS, BD sciences, San Jose, CA, USA) agar plates and incubated at 37 °C for 24 h. After colony formation, a single colony was transferred to 20 mL fresh MRS medium and incubated at 37 °C for 24 h to obtain a preculture suspension. To obtain *L. plantarum* metabolites, 1 mL of the precultured bacterial suspension was transferred to 40 mL of fresh MRS medium and incubated at 37 °C for 96 h. Thereafter, the suspension was centrifuged (3000× *g*, 10 min, 4 °C), and the supernatant was filtered using a syringe filter (0.2 μm pore size, Sartorius, Goettingen, Germany). The filtered *L. plantarum* metabolites (brown-colored clear solution) were stored at 4 °C until further use.

### 4.2. Cell Culture and Treatment

The human colonic epithelial cell line, Caco-2 (Cat. No: HTB-37TM), was obtained from the American Type Culture Collection (Manassas, VA, USA). Cells were cultured in high-glucose (4.5 g/L) Dulbecco’s Modified Eagle’s Medium containing 10% heat-inactivated fetal bovine serum (Hyclone Laboratories, Logan, UT, USA) without any antibiotics at 37 °C in a 5% CO_2_ atmosphere. The *L. plantarum* metabolites were diluted at various ratios: 0.5:99.5 (0.5%), 1:99 (1%), 2.5:97.5 (2.5%), 5:95 (5%), 7.5:92.5 (7.5%), 10:90 (10%), 12.5:87.5 (12.5%), 15:85 (15%), 17.5:82.5 (17.55), and 20:80 (20%). The diluted *L. plantarum* metabolites were also treated for 6 h, 9 h, 12 h, and 24 h at a fixed 10% dilution rate.

Caco-2 cells were plated in T-25 flasks at 2 × 10^6^ cells. The *L. plantarum* metabolites were diluted in media without antibiotics. CQ (Sigma-Aldrich, St. Louis, MO, USA) was diluted to a final concentration of 100 μM before use.

### 4.3. Cell Viability Assay

Caco-2 cells were treated with various concentrations of *L. plantarum* metabolites for 24 h. After incubation, 3-(4,5-dimethylthiazol-2-yl)-2,5-diphenyl tetrazolium bromide (MTT) solution (0.5 mg/mL, Sigma-Aldrich) was added to the cells, followed by incubation for 2 h at 37 °C in the dark. After forming purple formazan crystals, the spent medium was removed, and the cells were incubated with 100 μL of dimethyl sulfoxide until the purple formazan crystals were completely dissolved. The absorbance of each well was then measured at 570 nm using a Multiskan GO microplate spectrophotometer (Thermo Fisher Scientific, Waltham, MA, USA).

Cell viability was also investigated using microscopic imaging. Caco-2 cells were treated with the same concentrations of *L. plantarum* metabolites for 24 h. After incubation, the spent medium was removed, and the plate was observed and photographed using an MIC-Eyecam-3Mpixel Microscope Digital Camera (Eyecam, Clarity Medical Systems, Pleasanton, CA, USA). Scale bar, 100 μm. The total magnification of the images is 100×. The fraction of live cells was measured using Image J 1.47.

### 4.4. Western Blot Analysis

After treatment with *L. plantarum* metabolites for 24 h, total protein extracts from whole-cell lysates of Caco-2 cells were prepared in a protein extraction solution (ELPIS Biotech, Daejeon, Republic of Korea) containing a protease inhibitor (Sigma-Aldrich). The cell lysates were vortexed and incubated on ice for 1 h. The cell lysates were then centrifuged at 15,000× *g* for 20 min at 4 °C. Protein concentrations were measured using a Pierce BCA Protein Assay Kit (Thermo Fisher Scientific). The protein solutions were then boiled at 95 °C for 5 min, and equal amounts of protein were loaded and separated using a 10% sodium dodecyl sulfate-polyacrylamide gel. Proteins were transferred to polyvinylidene difluoride membranes using a 500 mA transfer membrane for 1 h. Membranes blocked with 5% skim milk in 1× Tris-buffered saline with Tween-20 buffer (TBS-T) for 1 h at room temperature (RT). After blocking, the membranes were incubated overnight at 4 °C with primary antibodies against the following molecules: autophagy-related 9A (Atg9A) (Cat. No: NB110-56893, 1:1000, Novus Biologicals, Englewood, CO, USA), autophagy-related 5 (Atg5) (Cat. No:12994, 1:1000, Cell Signaling Technology, Danvers, MA, USA), autophagy-related 16-like 1 (Atg16L1) (Cat. No: NB110-82384, 1:1000; Novus Biologicals), Beclin-1 (Cat. No:3945, 1:1000, Cell Signaling Technology), lipidated form of microtubule-associated protein 1A/1B-light chain 3 I/II (LC3 I/II) (Cat. No:12741, 1:1000; Cell Signaling Technology), and β-actin (Cat. No: A5441, 1:10,000, Sigma-Aldrich). The membranes were washed seven times with TBS-T and incubated with horseradish peroxidase-conjugated goat anti-rabbit antibody (1:10,000; Enzo Life Sciences, Farmingdale, NY, USA) for 2 h at RT. Finally, the proteins were detected using an enhanced chemiluminescence detection system (WesternBrightTM ECL, Advansta, Menol Park, CA, USA). Original images for blots are published in Supplementary Materials.

### 4.5. TMRE Staining

Caco-2 cells were seeded at a density of 2 × 10^5^ cells in 35 mm confocal dishes. The cells were then treated with *L. plantarum* metabolites for 6 or 24 h. After incubation, the cells were treated with 0.05 μM TMRE (Cayman Chemical Company, Ann Arbor, MI, USA) for 20 min at 37 °C. After incubation, the cells were washed with cold phosphate-buffered saline (PBS) (pH 7.5) (Hyclone Inc., Logan, UT, USA). Images were obtained using a Nikon ECLIPSE TE 2000-U microscope (Nikon, Tokyo, Japan).

## Data Availability

Not applicable.

## Supplementary Materials

The following supporting information can be downloaded at: https://www.mdpi.com/article/10.3390/molecules28041890/s1, Figure S1: Western blot analysis of Atg5, Atg9A, Atg16L1, Beclin-1, and LC3I/II proteins in Caco-2 cells treated with *L. plantarum*; Figure S2: Western blot analysis of Atg5, Atg9A, Atg16L1, Beclin-1, and LC3I/II proteins in Caco-2 cells treated with *L. plantarum* and CQ.
