# Supplementary figures and images for: Lactobacillus plantarum Metabolites Elicit Anticancer Effects by Inhibiting Autophagy-Related Responses
Source: Molecules. 2023 Feb 16;28(4):1890. doi: 10.3390/molecules28041890 (PMC9966080; doi:10.3390/molecules28041890)

# Supplementary Materials

Figure S1.

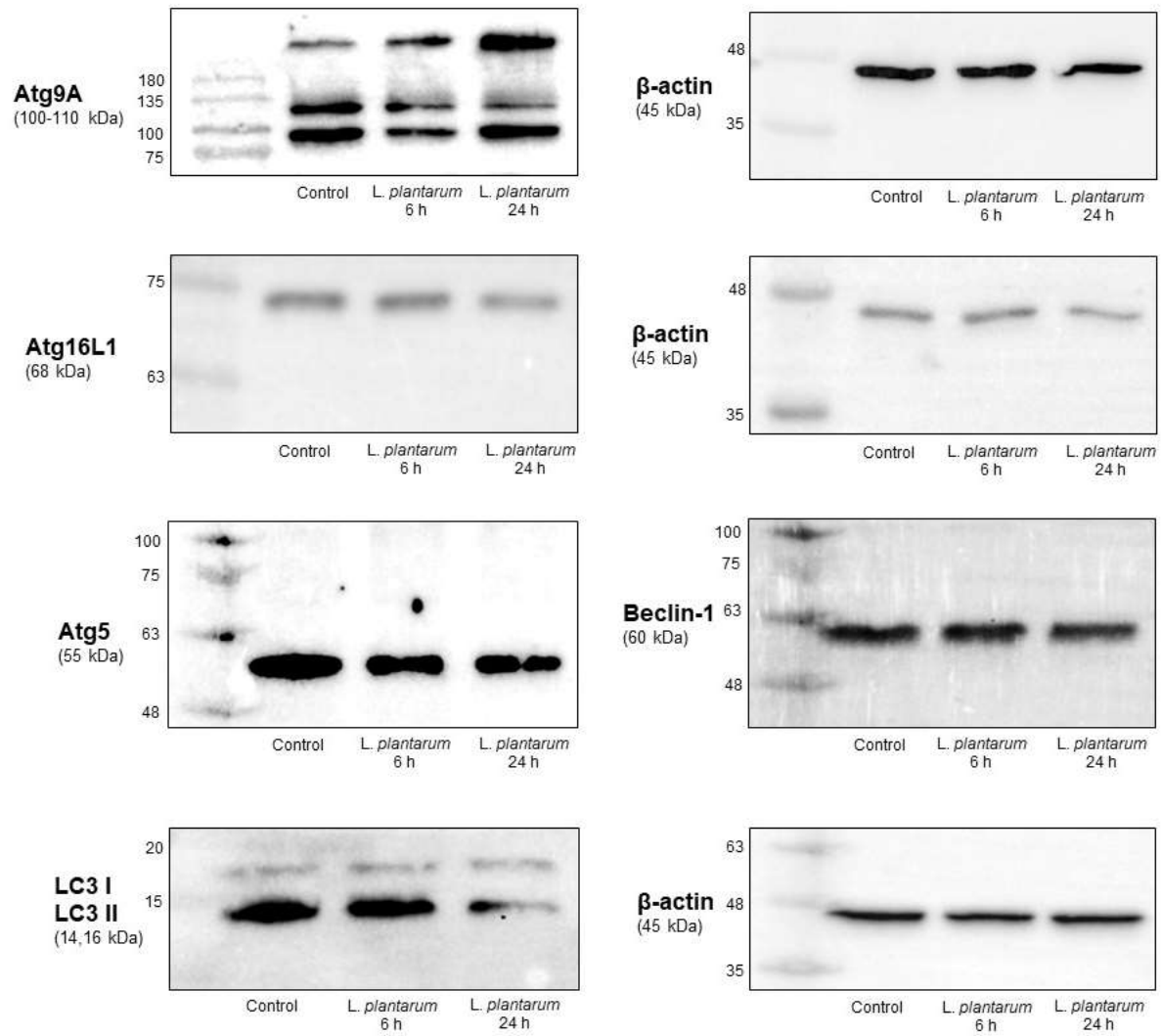

Figure S2.

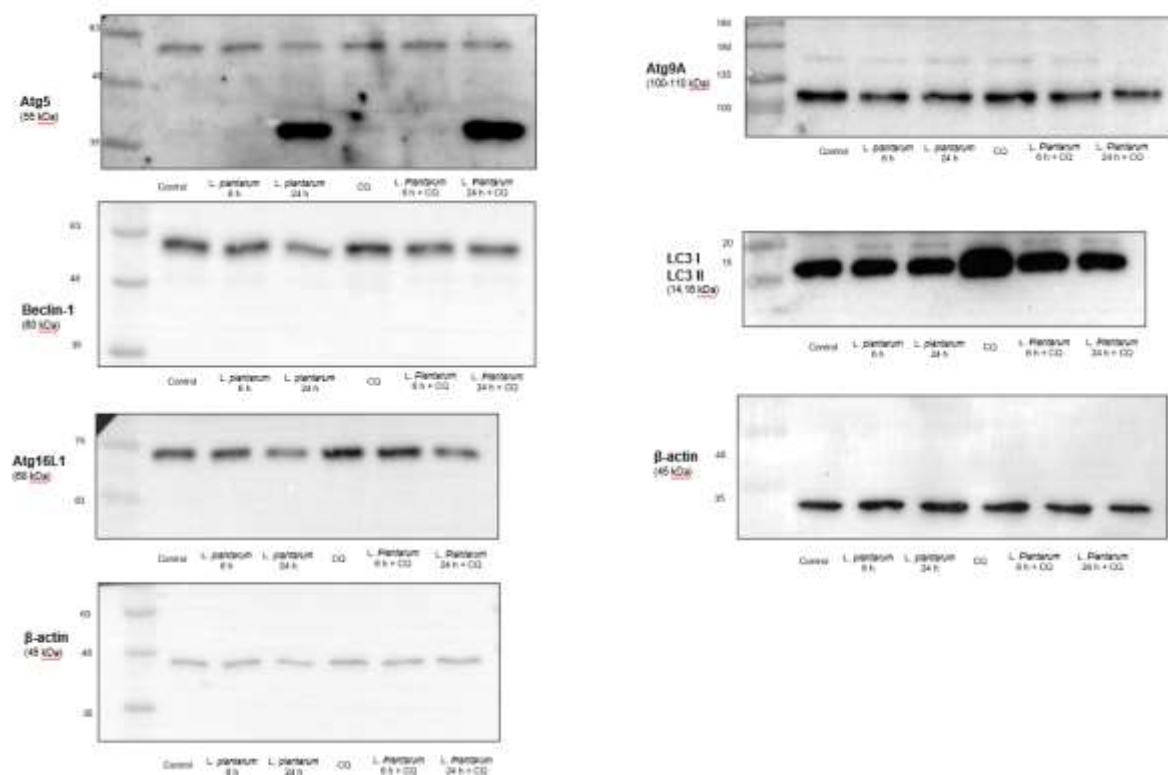

Supplement: Supplementary file 1 [file molecules-28-01890-s001.zip › molecules-2217649-supplementary.pdf]
